# Glioma-related seizures in relation to histopathological subtypes: a report from the glioma international case–control study

**DOI:** 10.1007/s00415-018-8857-0

**Published:** 2018-04-23

**Authors:** Shala G. Berntsson, Ryan T. Merrell, E. Susan Amirian, Georgina N. Armstrong, Daniel Lachance, Anja Smits, Renke Zhou, Daniel I. Jacobs, Margaret R. Wrensch, Sara H. Olson, Dora Il’yasova, Elizabeth B. Claus, Jill S. Barnholtz-Sloan, Joellen Schildkraut, Siegal Sadetzki, Christoffer Johansen, Richard S. Houlston, Robert B. Jenkins, Jonine L. Bernstein, Rose Lai, Sanjay Shete, Christopher I. Amos, Melissa L. Bondy, Beatrice S. Melin

**Affiliations:** 1 Department of Neuroscience, Neurology, Uppsala University, 751 85 Uppsala, Sweden; 2 Department of Neurology, NorthShore University HealthSystem, Evanston, IL USA; 3 Division of Medicine, Dan L. Duncan Cancer Center, Baylor College of Medicine, Houston, TX USA; 4 Department of Neurology, Mayo Clinic Comprehensive Cancer Center, Mayo Clinic, Rochester, MN USA; 5 Department of Neurological Surgery, University of California, San Francisco, San Francisco, CA USA; 6 Department of Epidemiology and Biostatistics, Memorial Sloan-Kettering Cancer Center, New York, NY USA; 7 Department of Epidemiology and Biostatistics, Georgia State University School of Public Health, Atlanta, Georgia; 8 Department of Epidemiology and Public Health, Yale University School of Medicine, New Haven, CT USA; 9 Case Comprehensive Cancer Center, Case Western Reserve University School of Medicine, Cleveland, OH USA; 10 Department of Public Health Sciences, University of Virginia, Charlottesville, VA USA; 11 Cancer and Radiation Epidemiology Unit, Gertner Institute, Chaim Sheba Medical Center, Tel Hashomer, Israel; 12 Sackler School of Medicine, Tel-Aviv University, Tel-Aviv, Israel; 13 Institute of Cancer Epidemiology, Danish Cancer Society, Copenhagen, Denmark; 14 Rigshospitalet, University of Copenhagen, Copenhagen, Denmark; 15 Section of Cancer Genetics, Institute of Cancer Research, Sutton, London, Surrey UK; 16 Department of Laboratory Medicine and Pathology, Mayo Clinic Comprehensive Cancer Center, Mayo Clinic, Rochester, MN USA; 17 Departments of Neurology, Neurosurgery, and Preventive Medicine, The University of Southern California Keck School of Medicine, Los Angeles, CA USA; 18 Department of Epidemiology, The University of Texas MD Anderson Cancer Center, Houston, TX USA; 19 Department of Radiation Sciences Oncology, Umeå University, Umeå, Sweden; 20 Department of Clinical Neuroscience, Institute of Neuroscience and Physiology, Sahlgrenska Academy, University of Gothenburg, Gothenburg, Sweden

**Keywords:** Observational study (cohort, case–control), Epileptic seizures, Primary brain tumor, Glioma-related seizures

## Abstract

**Background:**

The purpose of this study was to evaluate the distribution of glioma-related seizures and seizure control at the time of tumor diagnosis with respect to tumor histologic subtypes, tumor treatment and patient characteristics, and to compare seizure history preceding tumor diagnosis (or study enrollment) between glioma patients and healthy controls.

**Methods:**

The Glioma International Case Control study (GICC) risk factor questionnaire collected information on demographics, past medical/medication history, and occupational history. Cases from eight centers were also asked detailed questions on seizures in relation to glioma diagnosis; cases (*n* = 4533) and controls (*n* = 4171) were also asked about seizures less than 2 years from diagnosis and previous seizure history more than 2 years prior to tumor diagnosis, including childhood seizures.

**Results:**

Low-grade gliomas (LGGs), particularly oligodendrogliomas/oligoastrocytomas, had the highest proportion of glioma-related seizures. Patients with low-grade astrocytoma demonstrated the most medically refractory seizures. A total of 83% of patients were using only one antiepileptic drug (AED), which was levetiracetam in 71% of cases. Gross total resection was strongly associated with reduced seizure frequency (*p* < 0.009). No significant difference was found between glioma cases and controls in terms of seizure occurring more than 2 years before diagnosis or during childhood.

**Conclusions:**

Our study showed that glioma-related seizures were most common in low-grade gliomas. Gross total resection was associated with lower seizure frequency. Additionally, having a history of childhood seizures is not a risk factor ***for developing glioma-related seizures or glioma.

## Introduction

Epileptic seizures are among the most common presenting symptom in patients with glioma [1]. Glioma-related seizures may occur at different time points during the course of a patients’ illness, and can present either as focal seizures with or without impairment of consciousness or evolving to bilateral, convulsive seizures [2, 3]. The etiology of glioma-related seizures is complex and not completely understood, but patients diagnosed with low-grade gliomas (LGGs) have a higher risk for developing medically refractory epileptic seizures than those with higher tumor grades (i.e., anaplastic glioma and glioblastoma) [1]. An imbalance between the excessive release of neuroexcitatory glutamate and impaired GABAergic inhibition in the microenvironment surrounding the tumor has been suggested as one possible mechanism in glioma-related seizure development [4–7].

Treatment with antiepileptic drugs (AEDs) is critical for care of glioma patients, and good seizure control is an essential factor for improved quality of life. First-generation AEDs, such as phenytoin, carbamazepine, and valproic acid, have largely been replaced by newer AEDs, such as levetiracetam, lacosamide, and lamotrigine [8]. These newer drugs are characterized by lower hepatic enzyme inducing properties, fewer drug–drug interactions, and more favorable tolerability profiles.

The goal of the current study was threefold: (1) evaluate the distribution of glioma-related seizures at the time of tumor diagnosis with respect to tumor- and patient-related characteristics; (2) assess epileptic seizure frequency in relation to AED therapy and glioma treatment (surgery and radiotherapy); (3) compare seizure history preceding tumor diagnosis for cases and study enrollment for controls.

## Materials and methods

### Study population

The Glioma International Case–Control study (GICC) recruited 4533 cases and 4171 controls from 14 centers in the US, Europe, and Israel. Details on the GICC study population and methodology have previously been published [9]. Cases were between 18 and 80 years old at diagnosis, were histologically confirmed, and were recruited within 1 year of diagnosis of glioma.

Supratentorial and infratentorial gliomas were classified according to the 2007 WHO classification of brain tumors as follows: fibrillary astrocytoma (9420/3), protoplasmic astrocytoma (9410/3), gemistocytic astrocytoma (9411/3), oligodendroglioma (9450/3), oligoastrocytoma (9382/3), anaplastic astrocytoma (9401/3), anaplastic oligodendroglioma (9451/3), anaplastic oligoastrocytoma (9382/3), gliosarcoma (9442/3), or glioblastoma (9440/3) [10]. WHO grade I gliomas, which are usually low-proliferation, often-curable tumors were not included in this study. Thus, low-grade glioma (LGG) refers to WHO grade II tumors, including the following subtypes: astrocytoma, oligodendroglioma, and oligoastrocytoma. Anaplastic glioma refers to WHO grade III gliomas, including astrocytomas, oligodendrogliomas, and oligoastrocytomas, whereas glioblastoma refers to WHO grade IV glioma, the most malignant type included in this study. A pathology review was performed on participants in the first year of the study and demonstrated good concordance of the histopathology [9].

All recruitment sites received Institutional Review Board (IRB) or ethical board approval to conduct the study, and informed consent was obtained from participants.

### Data collection

Every site administered a common study protocol and questionnaire (full or abbreviated version), and data were stored in a centralized web-based database. The questionnaire was only administered at one time point. The GICC risk factor questionnaire collected information on demographic characteristics, past medical/medication history, and occupational history. Questionnaires were administered in person and/or by phone, or through mailed self-administered forms. Eight sites administered the full version of the questionnaire (The University of Texas MD Anderson Cancer Center, Danish Cancer Society Research Centre, Memorial Sloan Kettering Cancer Center, Brigham and Women’s Hospital, Case Western Reserve University, Mayo Clinic, NorthShore University HealthSystem and Umeå University). Some sites (Duke University, University of California, San Francisco, Institute of Cancer Research, UK, Gertner Institute, Israel, Columbia University, and University of Southern California) opted not to collect detailed seizure history [9]. All cases were asked if they had ever been diagnosed with seizures, convulsions, or epilepsy and the age at which they were diagnosed. In addition, at the sites that administered the full questionnaire, the glioma cases were asked if they had childhood seizures (febrile or not), when their epilepsy was diagnosed in relation to their brain tumor, if they had ever had an impairment of consciousness during a seizure, or bilateral convulsive epileptic seizures (grand mal), how many seizures they had had in the last 2 months, and if they were currently taking AED, and if so, what type of AED. For our analysis, we defined glioma-related seizures as seizures that began ≤ 2 years before diagnosis.

### Statistical analyses

The overall GICC analysis plan and a detailed table of demographics by study site are described elsewhere [9]. The main parameters of interest were history of seizures, the relative timing of the first epileptic seizure in relation to glioma diagnosis, age at epilepsy diagnosis, and epileptic seizure characteristics including impairment or loss of consciousness evolving to bilateral convulsive seizures, AED use, and seizure control (defined as recurrent epileptic seizures in spite of AED use and the subsequent frequency of seizures) during the last 2 months before the interview.

Case-only analyses were conducted using multinomial models. For the case–control analyses, we calculated adjusted odds ratios (ORs), along with their corresponding 95% Wald confidence intervals (CIs), using unconditional logistic regression. We decided a priori to control for age and sex, glioma subtype, and study site in all multivariable models. Despite our large sample size, some strata were sparse within the histologic subtypes (Table 2). As a result, we pooled the data from all sites rather than conducting meta-analysis. While we acknowledge that pooling has limitations because of the inter-site heterogeneity present in our consortium, the pooled analyses are exploratory, and therefore, allowed us to evaluate whether there might be some implication of an effect that should be examined in future studies of larger subgroups. Sensitivity analyses included running models with and without proxy responses (~ 8% of the cases).

## Results

Descriptive statistics for glioma cases (overall and by histologic subtypes) and controls are presented in Table 1. Median time interval from diagnosis to questionnaire was 3.2 months for GBM, 4.6 months for anaplastic astrocytoma and oligodendroglioma and finally 4.2 months for patients with low-grade gliomas.

**Table 1:** Selected population characteristics from the glioma international case–control study by case–control status and glioma histology group

	Glioma cases	Controls	Glioblastoma	Anaplastic astrocytoma	Anaplastic oligodendroglioma/oligoastrocytoma	LGG Astrocytoma	LGG Oligodendroglioma/oligoastrocytoma	Other
	*N* (%)	*N* (%)	*N* (%)	*N* (%)	*N* (%)	*N* (%)	*N* (%)	*N* (%)
Sex								
Male	2679 (59.1)	2351 (56.37)	1727 (62.3)	294 (55.58)	142 (51.64)	199 (53.35)	241 (53.44)	76 (57.14)
Female	1854 (40.9)	1820 (43.63)	1045 (37.7)	235 (44.42)	133 (48.36)	174 (46.65)	210 (46.56)	57 (42.86)
Age at diagnosis/enrollment								
18–29 years	308 (6.79)	294 (7.05)	62 (2.24)	68 (12.85)	25 (9.09)	65 (17.43)	70 (15.52)	18 (13.53)
30–39 years	521 (11.49)	473 (11.34)	108 (3.9)	115 (21.74)	54 (19.64)	99 (26.54)	122 (27.05)	23 (17.29)
40–49 years	813 (17.94)	680 (16.3)	417 (15.04)	110 (20.79)	63 (22.91)	89 (23.86)	114 (25.28)	20 (15.04)
50–59 years	1150 (25.3)	1079 (25.87)	795 (28.68)	90 (17.01)	64 (23.27)	67 (17.96)	98 (21.73)	36 (27.07)
60–69 years	1239 (27.3)	1098 (26.32)	993 (35.82)	94 (17.77)	52 (18.91)	40 (10.72)	37 (8.2)	23 (17.29)
70–80 years	502 (11.07)	547 (13.11)	397 (14.32)	52 (9.83)	17 (6.18)	13 (3.49)	10 (2.22)	13 (9.77)
Seizure history^a^								
Glioma-related seizures (seizures ≤ 2 years of diagnosis)	1158 (28.5)	–	527 (21.11)	186 (39.74)	84 (36.68)	150 (43.35)	188 (45.30)	23 (19.83)
Non-glioma-related seizures (seizure initiation started > 2 years before diagnosis	106 (2.60)	–	55 (8.86)	15 (6.79)	2 (2.15)	13 (7.22)	14 (6.20)	7 (20.00)
Cases with seizures where time of seizure initiation not reported	112 (2.75)	–	39 (6.28)	20 (9.05)	7 (7.53)	17 (9.44)	24 (10.62)	5 (14.29)
No seizures	2568 (63.1)	–	1792 (71.79)	233 (49.79)	130 (56. (77)	157 (45.38)	179 (43.13)	77 (66.38)
Missing	126 (3.1)	–	83 (3.33)	14 (2.99)	6 (2.62)	9 (2.6)	10 (2.41)	4 (3.45)

*LGG* low-grade glioma

^a^UK did not answer this section and was excluded. ORs adjusted for sex and age

3944 of the 4533 (87%) glioma cases and 3244 of the 4171 (78%) controls completed the full questionnaire (Table 5). Glioma occurred more frequently in males than females (59 vs. 41%), both in the entire sample and by tumor subtypes. Patients with LGG were younger at diagnosis (30–39 years) than patients with anaplastic glioma (40–59 years) or glioblastoma (50–69 years). Approximately, one-third of all glioma cases (28.5%) reported diagnosis of seizures, convulsions, or epilepsy (Table 1). Among cases reporting the relative timing of their first seizures (*n* = 1376), there were cases who, reported uncertainty about the time interval (*n* = 112), leaving a total of 1264 cases for the analysis. Epileptic seizures occurred most commonly within 2 years prior to glioma diagnosis (*n* = 1158) (Table 1).

There was a significant difference in history of ever having been diagnosed with epileptic seizures (including seizure diagnoses within 2 years prior to tumor diagnosis) between patients with glioblastoma (26%) and those with low-grade oligodendroglioma/oligoastrocytoma (56%) or low-grade astrocytoma (53%) (Table 2). Grade II oligodendroglioma/oligoastrocytoma patients were 3 times more likely to have had a history of epileptic seizures compared to glioblastoma patients (OR = 3.03; CI 2.41–3.82, *p *< 0.0001) (Table 2).

**Table 2:** Characteristics of seizures in glioma cases according to histology subtype

	Glioblastoma [Ref]	ORs	Anaplastic astrocytoma		Anaplastic oligo/oligoastrocytoma		LGG astrocytoma		LGG oligo/oligoastrocytoma		Other	Trend *p* value
	*N* (%)		*N* (%)	OR (95% CI)	*N* (%)	OR (95% CI)	*N* (%)	OR (95% CI)	*N* (%)	OR (95% CI)	*N* (%)	
Ever diagnosed with seizure, convulsions, or epilepsy^a^												< 0.0001
No [ref]	1792 (74.3)	Ref	233 (51.3)		130 (58.3)		157 (46.6)		179 (44.2)		77 (68.8)	
Yes	621 (25.7)	Ref	221 (48.7)	2.44 (1.98–3.02)	93 (41.7)	1.84 (1.38–2.45)	180 (53.4)	2.77 (2.17–3.54)	226 (55.8)	3.03 (2.41–3.82)	35 (31.3)	
Ever lost consciousness while having a seizure^b^												< 0.0001
No [ref]	658 (71.8)	Ref	132 (56.9)		74 (61.7)		83 (49.1)		119 (51.7)		30 (60.0)	
Yes	258 (28.2)	Ref	100 (43.1)	1.53 (1.12–2.08)	46 (38.3)	1.30 (0.87–1.95)	86 (50.9)	1.89 (1.33–2.69)	111 (48.3)	1.62 (1.18–2.23)	20 (40.0)	
Currently taking medications including prevention^b^												< 0.0001
No [ref]	690 (51.3)	Ref	100 (34.5)		59 (39.1)		60 (27.9)		87 (33.6)		29 (42.0)	
Yes	655 (48.7)	Ref	190 (65.5)	1.63 (1.24–2.15)	92 (60.9)	1.35 (0.95–1.92)	155 (72.1)	1.97 (1.42–2.75)	172 (66.4)	1.46 (1.08–1.97)	40 (58.0)	
Recurrent seizure in spite of medication^c^												0.1462
No [ref]	355 (69.5)	Ref	115 (69.7)		57 (73.1)		80 (56.7)		101 (63.1)		23 (71.9)	
Yes	156 (30.5)	Ref	50 (30.3)	0.90 (0.60–1.34)	21 (26.9)	0.78 (0.45–1.34)	61 (43.3)	1.55 (1.03–2.33)	59 (36.9)	1.17 (0.78–1.75)	9 (28.1)	

Oligo is oligodendroglioma. LGG is low-grade Glioma

^a^UK did not answer this section and was excluded. ORs adjusted for sex and age and site

^b^Duke, UCSF, UK, Israel, Columbia and USC did not answer this section and were excluded. ORs adjusted for sex and age

^c^Duke, UCSF, UK, Israel, Columbia and USC did not answer this section and were excluded

Patients with grade II oligodendroglioma/oligoastrocytoma, grade II astrocytoma, and anaplastic astrocytoma were more likely to have generalized epileptic seizures than cases with glioblastoma and anaplastic oligodendroglioma *(p* < 0.0001) (Table 2). There were a higher proportion of LGG patients who had recurrent epileptic seizures than cases with anaplastic glioma and glioblastoma, despite treatment with AEDs. Low-grade astrocytoma cases were about 1.6 times more likely to have recurrent epileptic seizures compared to glioblastoma cases (OR = 1.55; 95% CI 1.03–2.33 *p* = 0.15) (Table 2).

Overall, 83% of cases with epileptic seizures were prescribed one AED; 12% were on two AEDs; and 3% used 3–5 AEDs. Levetiracetam was the AED of choice in 71% of cases (Table 3).

**Table 3:** Seizure information and use of medication for seizure in glioma cases) by glioma histology group

	Glioblastoma *N* (%)	Anaplastic astrocytoma *N* (%)	Anaplastic oligo/oligoastrocytoma *N* (%)	LGG astrocytoma *N* (%)	LGG oligo/oligoastrocytoma *N* (%)	Other *N* (%)	Total *N* (%)
As a child ever had seizures with high fever^b^							
Yes	22 (1.53)	10 (3.27)	2 (1.24)	4 (1.79)	4 (1.5)	1 (1.37)	43 (1.74)
No	1365 (94.92)	285 (93.14)	158 (98.14)	212 (94.64)	253 (94.76)	68 (93.15)	2341 (94.82)
Missing	51 (3.55)	11 (3.59)	1 (0.62)	8 (3.57)	10 (3.75)	4 (5.48)	85 (3.44)
Has a child ever had seizure not caused by high fever^b^							
Yes	18 (1.25)	5 (1.63)	0	4 (1.79)	5 (1.87)	4 (5.48)	36 (1.46)
No	1374 (95.55)	290 (94.77)	156 (96.89)	216 (96.43)	252 (94.38)	65 (89.04)	2353 (95.3)
Missing	46 (3.2)	11 (3.59)	5 (3.11)	4 (1.79)	10 (3.75)	4 (5.48)	80 (3.24)
Ever had a bilateral convulsive seizure^b^							
Yes	124 (28.18)	59 (35.33)	31 (37.8)	52 (36.36)	61 (36.53)	13 (41.94)	340 (33.01)
No	245 (55.68)	85 (50.9)	37 (45.12)	69 (48.25)	79 (47.31)	15 (48.39)	530 (51.46)
Missing	71 (16.14)	23 (13.77)	14 (17.07)	22 (15.39)	27 (16.17)	3 (9.68)	160 (15.54)
No of seizure medications^a^							
1	339 (85.18)	126 (80.77)	68 (89.47)	104 (77.61)	120 (79.47)	23 (88.46)	780 (82.89)
2	44 (11.06)	23 (14.74)	5 (6.58)	21 (15.67)	20 (13.25)	3 (11.54)	116 (12.33)
3	5 (1.26)	3 (1.92)	1 (1.32)	8 (5.97)	8 (5.3)	0	25 (2.66)
4	0	1 (0.64)	0	1 (0.75)	1 (0.66)	0	3 (0.32)
5	1 (0.25)	0	0	0	0	0	1 (0.11)
Missing	9 (2.26)	3 (1.92)	2 (2.63)	0	2 (1.32)	0	16 (1.7)
Ever taken levetiracetam^b^							
Yes	301 (75.63)	112 (71.79)	49 (64.47)	90 (67.16)	98 (64.9)	18 (69.23)	668 (70.99)
No	97 (24.37)	44 (28.21)	27 (35.53)	44 (32.84)	53 (35.1)	8 (30.77)	273 (29.01)

Oligo is oligodendroglioma, LGG is low-grade glioma

^a^MDA, Denmark, MSK, Brigham and Women, Case Western, Mayo, Northshore and Sweden provided this information

^b^Duke, UCSF, UK, Israel, Columbia and USC did not ask these questions

Treatment information in terms of type of surgery (gross total resection vs. subtotal resection/biopsy) and radiotherapy (yes or no) was only available in a subset of cases (23%). Gross total resection (GTR) was strongly associated with reduced frequency of epileptic seizures; glioma cases who had GTR were 72% less likely to have reported > 10 seizures during the 2 months immediately prior to the interview, compared with cases that underwent subtotal resection or biopsy (*n* = 255) (OR = 0.28; CI 0.11–0.73, *p* < 0.009). Radiotherapy, during first-line treatment, was not associated with epileptic seizure control (*p* < 0.85) (Table 4).

**Table 4:** Association between seizure frequency during the last 2 months and glioma case subtype

	Seizure free; ref; *N* (%)	1–5 seizures *N* (%)	6–10 seizures *N* (%)	> 10 seizures *N* (%)	Total *N*	1–5 seizures OR (95% CI)	6–10 seizures OR (95% CI)	> 10 seizures OR (95% CI)	*P* value
Glioma subtype									0.7461
Glioblastoma [ref]	380 (80.34)	70 (14.80)	6 (1.27)	17 (3.59)	473	1.00	1.00	1.00	
Anaplastic oligo/oligoastrocytoma	51 (78.46)	10 (15.38)	1 (1.54)	3 (4.62)	65	0.95 (0.45–2.00)	0.82 (0.09–7.48)	1.14 (0.31–4.16)	
Anaplastic astrocytoma	92 (74.80)	24 (19.51)	1 (0.81)	6 (4.88)	123	1.24 (0.72–2.14)	0.42 (0.05–3.87)	1.22 (0.45–3.30)	
LGG, astrocytoma	65 (70.65)	13 (14.13)	3 (3.26)	11 (11.96)	92	0.90 (0.44–1.85)	1.86 (0.36–9.61)	3.25 (1.27–8.29)	
LGG, oligo/oligoastrocytoma	87 (72.50)	23 (19.17)	3 (2.50)	7 (5.83)	120	1.16 (0.60–2.23)	1.35 (0.23–7.78)	1.47 (0.49–4.39)	
Other	22 (78.57)	3 (10.71)	1 (3.57)	2 (7.14)	28	0.63 (0.18–2.22)	1.93 (0.19–19.67)	1.73 (0.35–8.40)	
Sex									0.1522
Male [ref]	411 (78.74)	84 (16.09)	4 (0.77)	23 (4.41)	522	1.00	1.00	1.00	
Female	286 (75.46)	59 (15.57)	11 (2.90)	23 (6.07)	379	0.96 (0.67–1.40)	3.54 (1.10–11.39)	1.31 (0.71–2.41)	
Gross total resection									0.009
No [ref]	481 (74.46)	111 (17.18)	13 (2.01)	41 (6.35)	646	1.00	1.00	1.00	
Yes	216 (84.71)	32 (12.55)	2 (0.78)	5 (1.96)	255	0.64 (0.42–0.99)	0.36 (0.08–1.64)	0.28 (0.11–0.73)	
Radiotherapy									0.8558
No [ref]	149 (73.40)	36 (17.73)	4 (1.97)	14 (6.90)	203	1.00	1.00	1.00	
Yes	548 (78.51)	107 (15.33)	11 (1.58)	32 (4.58)	698	0.83 (0.50–1.38)	1.17 (0.30–4.64)	0.82 (0.38–1.76)	
Mean age at glioma diagnosis	52.7	51.2	46.7	50.3		0.99 (0.98–1.01)	0.98 (0.94–1.02)	1.00 (0.98–1.03)	0.6138
Total	697	143	15	46					

Duke, UCSF, UK, Israel, Columbia, and USC did not take part in this section and were excluded. ORs adjusted for sex, age, gross total resection status (yes/no) and radiotherapy within 6 months (yes/no)

Glioma cases were as likely as controls to experience febrile (OR = 1.13; 95% CI 0.73–1.75, *p* = 0.59) and non-febrile seizures (OR = 1.61; 95% CI 0.93–2.76, *p* = 0.09) during childhood (Table 5).

**Table 5:** Association between seizure diagnosis and case–control status

	Case *N* (%)	Control *N* (%)	Multivariate models ORs (95% CI)	*P* value
Ever diagnosed with seizure^a^				< 0.0001
No [ref]	2568 (65.11)	3141 (93.82)	1.00	
Yes	1376 (34.89)	103 (3.18)	18.97 (15.32–23.48)	
Non-glioma-related seizures (seizure initiation started > 2 years before diagnosis)^a^				0.221
No [ref]	3838 (97.31)	3141 (96.82)	1.00	
Yes	106 (2.69)	103 (3.18)	0.93 (0.69–1.24)	
Febrile seizures as a child^b^				0.5944
No [ref]	2341 (98.20)	2413 (98.29)	1.00	
Yes	43 (1.80)	42 (1.71)	1.13 (0.73–1.75)	
Non-febrile seizure as a child				0.0874
No [ref]	2353 (98.49)	2474 (99.08)	1.00	
Yes	36 (1.51)	23 (0.92)	1.61 (0.93–2.76)	

^a^UK did not answer this section and was excluded. ORs adjusted for sex, glioma subtype, diagnosis age, and study sites

^b^Duke, UCSF, UK, Israel, Columbia and USC did not answer this section and were excluded. *Ref*  reference condition. Those with missing data were excluded from OR calculations

## Discussion

Our study is one of the largest studies to date of glioma-related seizures in relation to proximity of diagnosis, glioma histology, and treatment by AEDs, as well as tumor treatment by resection and radiotherapy. Our results confirms a lower mean age at onset in patients with LGGs compared to anaplastic glioma and glioblastoma, as well as a higher rate of epileptic seizures as a presenting symptom, a phenomenon that has been well established in other large epidemiological studies [11–13]. We also observed a higher frequency of seizures among low-grade oligodendroglioma/oligoastrocytoma cases compared to glioblastoma cases, consistent with previous reports. Our data confirm the relatively long time interval that is needed for the development of tumor-related seizures in the brain and the association between seizure risk and the growth rate of the tumor. Patients with LGGs, and particularly oligodendroglial tumors, are therefore, generally more prone to seizures than patients with high-grade gliomas [3, 14, 15]. The mechanisms behind seizure development in slow-growing tumors are different from high-grade tumors. Chronic deafferentation and disconnection of functionally isolated regions of cortex, causing a denervation hypersensitivity is connected to seizure risk in LGGs, whereas the direct effects of tissue damage in fast-growing high-grade gliomas by disturbed microvascularization and peritumoral ischemia are thought to be causative factors [16, 17].

We also found that patients with low-grade astrocytoma patients had significantly more recurrent seizures in spite of combined antiepileptic treatments. These differences in seizure control may reflect the differences in tumor locations of the glioma subtypes. Astrocytomas are more frequently associated with location in temporal or insular areas, while oligodendroglial tumors are more often located in frontal areas [14, 18].

Refractory seizures in patients with LGGs is a major concern in clinical neuro-oncology practice [19]. The epileptogenic zone of LGGs, especially in tumors located in temporal and paralimbic areas, involves not only the tumor itself but also extra-tumoral cortical areas, explaining the poor seizure control in 15–20% of patients after gross total tumor resection. Improved postoperative seizure control was achieved in cases where the resection involved both the tumor and the epileptogenic zone surrounding the tumor [20–24].

Moreover, the treatment of glioma-related epilepsy with AED is complex. Cognitive side effects, interactions between AEDs and between AED treatment and chemotherapy are important aspects that need to be considered. Increased susceptibility to the cognitive side effects of AEDs was much more frequent than the side effects of radiotherapy in patients with LGGs [25]. Finally, the difficulties of obtaining good seizure control in spite of optimal AED and tumor treatment also illustrate the natural course of LGGs as a progressive and eventually fatal disease.

Our data demonstrate that patients with LGG, particularly low-grade astrocytoma, should be closely monitored for seizures.

Recently, a large multicenter French study provided detailed information on approximately 1500 LGGs, and identified male sex and tumor location in functional areas as independent predictors for tumor-related epileptic seizures [12]. The correlation between glioma-related seizures and male sex was not confirmed in the present study. In addition, among LGGs, the prevalence of self-reported glioma-related seizures was slightly lower in our study than in previous studies [1, 12, 14, 26]. A potential reason is the possible poor recall of events. Patients may only report more dramatic seizure episodes, such as secondary generalized seizures, and due to cognitive decline, may fail to recall less severe focal seizures, such as left-hemispheric temporal lobe seizures. In our study, data were collected through self-reported questionnaires, as opposed to clinical observation or medical record abstraction, which could be one of the reasons for differences in the rates of epileptic seizures between the studies.

Furthermore, LGGs and anaplastic astrocytomas were more commonly associated with generalized seizures than glioblastoma. These findings support the existence of different epileptogenic pathways for LGG and high-grade gliomas [17, 24]. From an epileptogenic point of view, this finding reinforces the general concept that slow-growing tumors have a lower epileptic threshold than fast-growing lesions [1].

Somatic isocitrate–dehydrogenase 1 (IDH1) mutations are common in LGGs, leading to the production of D-2 hydroxyglutarate, a metabolite that bears structural similarities to glutamate [27]. Thus, the IDH1 mutation in the tumor cells renders the tumor in a higher excitatory state and may be directly involved in the pathogenesis of tumor-related seizures in LGGs [28]. Routine testing of IDH1 mutational status was not performed at the time of our data collection. However, given that the frequency of IDH mutation in LGG approaches 80% and its possible role in epileptogenesis, a partial explanation for the observed higher frequency of epileptic seizures in LGGs could be due to IDH1 mutation status [29].

In this study, we only have data from time of study enrollment/diagnosis, and no follow-up data was collected. Glioma patients who presented with other tumor-related symptoms may not have an initial seizure until much later in course of their disease. Thus, these cases may not have been recognized as tumor-related seizures occurring post-glioma diagnosis.

Levetiracetam was the most commonly used AED to treat patients in our study. Levetiracetam is a newer generation AED that has predictable pharmacokinetics with less concern for interaction with chemotherapeutic drugs, as is the case with the first-generation enzyme-inducing drugs, phenytoin and carbamazepine [30]. Newer generation AEDs such as levetiracetam do not induce or inhibit hepatic P450 enzymes. For this reason, the first-generation AEDs are often prohibited from being used in glioma patients who are enrolled in clinical trials. Although not more effective than first-generation AEDs, levetiracetam has been used as monotherapy with efficacy of 70–100% in gliomas, and is one of the drugs of choice for glioma-related seizures due to its favorable tolerability profile [28, 31, 32]. However, the standard of care for epileptic seizure prophylaxis may be different between the recruitment sites.

Although we only had data from a subset of the population, which may not be representative of the population as a whole, we observed a significant correlation between reduced seizure frequency and gross total resection, which is also consistent with previous studies where the extent of surgical resection is recognized as an independent predictor of controlled epileptic seizures after oncological treatment [14, 20, 33].

Based on the patient’s status and specific characteristics, however, maximal surgery may have to be postponed, particularly if the mass is situated in the eloquent areas of the brain.

Oncological treatment of the tumor is essential for glioma-related seizure control, and previous studies have reported improved seizure control in patients with LGGs after radiotherapy [34–36]. Moreover, two series of patients with low-grade gliomas have shown a significant lower seizure frequency following interstitial brachytherapy [37, 38].

However, we were not able to confirm this in our study, most likely because the interviews were conducted soon after the patient’s diagnosis (median 3.6 months post-diagnosis), when improvement of seizure control by radiotherapy may not yet be achieved.

There was no significant difference in the incidence of epileptic seizures > 2 years before diagnosis or in the history of seizures during childhood between cases and controls. A causal relationship between increased glioma risk and a history of epilepsy and/or exposure to AEDs long before brain tumor diagnosis has not been established [40–42]. Our study does not show that childhood seizures are risk factors for glioma-related seizures.

### Limitations of the study

This study was a retrospective analysis of the GICC epidemiological study and an extensive medical chart review was not part of the analysis. One limitation common to all case–control studies is the potential for recall bias; although in this study, it is more likely that cases under-reported seizures due to poor memory related to cognitive impairment. We also acknowledge that grouping data from different centers that may have different standards of care may introduce a potential bias when interpreting the results. Furthermore, the results are based entirely on a one-time questionnaire completion and some centers opted out of full questionnaire completion. Another limitation of our study is related to the lack of information on the exact location of the tumor and volume estimates at surgery, variables that have been associated with seizure risk and control in gliomas [12, 24]. Additionally, a significant limitation is that we did not have access to detailed oncological treatment data, as the primary aim of the GICC was to analyze potential epidemiological associations and gene-environment interactions. In addition to what has been reported for the effects of radiotherapy on seizure control, prior studies have found positive effects of chemotherapy on epileptic seizure control. Our study was not primarily aimed at looking at these clinical factors, so we have limited data on treatment-related variables with the possibility of potential bias in the reported conclusions.

Our cases were diagnosed prior to the 2016 WHO classification so they are not classified with IDH mutation and 1p/19q co-deletion status [43]. Future studies within the consortium will determine if there are genes that may lead to increased susceptibility to glioma-related seizures.

## Conclusion

In conclusion, our large case series demonstrates a pattern of epileptic seizure prevalence and seizure control in patients with gliomas that confirms previous reports. Our study also confirms the strong association between epileptic seizures and slowly growing tumors. No relationship was found between a history of febrile seizures and glioma-related epileptic seizures, suggesting that remote seizure may not be a risk factor for developing glioma-related seizures or glioma.
